# Cloth face mask fit and function for children part two: Material Selection

**DOI:** 10.1186/s40691-022-00315-7

**Published:** 2022-12-05

**Authors:** Katarina Goodge, Jenny Leigh Du Puis, Mona Maher, Margaret W. Frey, Fatma Baytar, Heeju Park

**Affiliations:** Department of Human Centered Design, College of Human Ecology, Cornell University, 37 Forest Home Dr., 235 Human Ecology Building, Ithaca, NY 14853-4401 USA

**Keywords:** Face mask, Children, COVID-19, Cloth mask materials, Reusable, Filtration efficiency, Air permeability

## Abstract

The second component of this three-paper series studying cloth face masks for children ages 4 to 6 years old concentrates on optimizing aerosol capture and air permeability through fabric selection. Material choices were evaluated in two modes: Flat Filter (FF) and Head Form (HF). FF isolates material factors while HF simulates the performance of the constructed masks on a 3D printed child head form. In FF mode, higher filtration efficiency correlated to lower air permeability in both reusable commercial and experimental face masks regardless of fiber contents, fabric structures, and number of layers. Our prototype face mask developed in design exploration successfully captured 37 ± 12% of 0.3 μm, 87 ± 3% of 0.5 μm, and 87 ± 2% of 1.0 μm particles while maintaining good air permeability, moisture capture, and aerosolized salt capture in HF mode. Sealing masks to minimize outward leakage reduced particle capture up to 64%. Particle leakage data captured at the eye level of the head form illuminated the synergy between mask design, material choice, and fit.

## Introduction

The COVID-19 pandemic provoked a surge in the everyday use of face masks for children as young as 2 years old to adults of any age (Centers for Disease Control and Prevention, n.d.). A critical lack of standardization or sizing for children’s masks coupled with children’s higher respiration rates and lower breathing pressure than adults put children’s face mask designers/manufacturers and children’s caregivers at a unique disadvantage when designing and choosing face masks. Cloth face masks act as a form of source control and offer a certain level of protection to the wearer, but are currently not regulated for medical use or as respiratory protection devices (Centers for Disease Control and Prevention, n.d.; Courtney & Bax, 2021). The American Association of Textile Chemists & Colorists (AATCC) released M14-2020 guidance document for nonmedical face coverings in mid-2020 and ASTM published F3502-21 standard for Barrier Face Coverings in early 2021 (American Association of Textile Chemists and Colorists, 2020; ASTM International, 2021). Both AATCC M14 and ASTM F3502 are for adults only.

A children's standard is important due to the physiological differences between adults and children's respiratory systems: respiratory rates decrease with age, so for children 4–6 years old the rate typically ranges from 22–34 breaths per minute whereas adults average 12–20 breaths per minute (Eleesha & Kirsten, 2021; New York State Department of Health, n.d.). Coupled with higher breathing frequencies, Szeinberg and colleagues indicated that children’s breathing pressure is lower than in adults (Szeinberg et al., 1987). Therefore, the same mask airflow resistance will put more stress on a child’s respiratory system than an adult’s system. Lower velocities at the fabric surface correlate to higher filtration efficiencies, but higher frequency of respiration decreases the filtration efficiency (Tcharkhtchi et al., 2021). Thus, one “best” material choice does not exist, and one mask tested under one set of specified conditions (adult breathing parameters) does not necessarily mean it will perform the same for a separate set of conditions (child breathing parameters).

Material choice is important in face mask design as it directly determines the breathability, particle capture, and comfort factors of the end-use product (Clase et al., 2020; Rogak et al., 2021). Particularly, discomfort was noted as a common reason for children to wear masks incorrectly in a survey conducted by Du Puis and colleagues (Du Puis et al., 2022). Limited research has been conducted on using commonly available fabrics in nonmedical face masks and included only adult breathing rates for testing parameters. To test materials for face mask use, filtration efficiency tests can be performed on flat filters (Konda et al., 2020; Rengasamy et al., 2010, 2017; Reyes & Frey, 2017; Zangmeister et al., 2020; Zhao et al., 2020) or fully constructed masks (Rengasamy et al., 2017). Filtration tests are typically performed under constant aerosol flow, but unsteady or cyclic flow patterns, similar to human breathing, result in higher penetration of particles (Tcharkhtchi et al., 2021). Thus, breathing simulators with attached head forms have been used to test children’s oxygen masks (Napolitano et al., 2017), simulation of human coughing or sneezing (Verma et al., 2020), source control (Patel et al., 2016), and total inward leakage (Rengasamy et al., 2014). While these studies demonstrate the effectiveness of the head form and breathing simulator as a mask testing tool, none have utilized this type of system to test children’s cloth face masks on a child-sized head form.

Therefore, the purpose of the present paper was to evaluate commonly used fabrics and layering combinations in children’s commercial face masks as well as potential alternatives. All fabrics were tested as flat textiles to isolate the material effect on filtration efficiency, air permeability, and aerosol capture. The potentially effective fabric combinations were further constructed into a mask design mimicking a commercial shaped mask and our prototype mask (Du Puis et al., 2022). These constructed masks alongside a single-use children’s procedure mask as a positive control were tested on a simulated breathing head form to assess the combination of material and design effects on filtration efficiency, air permeability, and aerosol capture.

The objectives of the present study were to answer the following research questions (RQ):

- RQ1: How do commercially available, reusable cloth face masks perform in terms of filtration efficiency and air permeability for children ages 4 to 6 years old?
- RQ2: How can reusable cloth face mask material choice be optimized for respiratory patterns of children ages 4 to 6 years old?
- RQ3: What interaction effect does reusable cloth face mask design, fit, and material choice have on filtration efficiency and air permeability?
  - RQ3a: How do the Flat Filter mode results compare to the Head Form mode results?
  - RQ3b: How do the sealed mask results compare to the unsealed mask results in Head Form mode? Do the sealed mask results approach the Flat Filter mode results?

## Methods

### Materials

#### Commercial face masks

Commercial masks, listed in Table 1, were chosen to reflect the range of shapes and material compositions available (for images of these masks, see Appendix).

**Table 1:** Material specifications of chosen commercial children face masks

ID	Layer	Fiber content	Fabric structure	Mean fabric weight (gsm)	Mean fabric thickness (mm)	Mean TPI (warp/wale, weft/courses)
A	Outer	80% Nylon, 20% Spandex	Jersey knit	221.70	0.72	71, 122
	Inner	96% Polyester, 4% Spandex	Jersey knit	118.07	0.43	52, 65
B	Single	100% Cotton	Plain weave	120.09	0.25	75, 150
C	Outer	100% Cotton	Plain weave	70.69	0.20	104, 80
	Mid	100% Polypropylene	Melt-blown nonwoven	175.03	0.38	N/A
	Inner	100% Cotton	Plain weave	–	0.45	61, 39
D	Single	Polyester*	Warp knit	272.17	0.65	48, 63
E	Single	90% Polyester, 10% Spandex	Jersey knit	183.26	0.56	45, 53
F	Outer	100% Polyester	Interlock	180.67	0.30	44, 49
	Inner	100% Polyester	Plain weave	71.66	0.13	83, 96
G	Outer	Polyester*	Warp knit	201.24	0.65	42, 50
	Inner	Cotton*	Jersey knit	164.30	0.51	47, 73
H	Single	Cotton*	Jersey knit	143.11	0.50	48, 67

*Identified following ASTM D276 Infrared Spectra Scheme

Each commercial mask was deconstructed into its individual layers to perform the material testing. Unless fiber content was specified by the product description or garment tag, it was identified following ASTM D276 Infrared Spectra Scheme. Fabrics were characterized for fabric weight (ASTM D3776-20 (Option C—Small Swatch of Fabric)), warp and weft thread count (ASTM D3775-17e1 (woven) and D8007-15(2019) (knit)), thickness (ASTM D1777-96(2019)), and air permeability (ASTM D737-18) following standard testing methods adjusted to accommodate three replicates within the small swatches taken from the deconstructed pieces of each face mask. Descriptive statistics were calculated as the mean and standard error throughout. All fabrics were conditioned according to ASTM D1776M-20 for at least 24 h before testing.

For filtration and air permeability tests, multilayer samples were assembled in the sequence of the original mask and tested as the composite. The assembly was oriented with the Inner layer facing the inlet of the filter holder to mimic the passage of exhaled breath through the donned face mask. Masks that only had two layers were labeled as “Inner Layer” and “Outer Layer”. Single layer masks were labeled as “Inner Layer”. Mid and Inner layers were glued together in Mask C and weighed as such.

#### Experimental face masks

Fabric for the experimental face masks were nominated based on their air permeability (Goodge & Frey, 2021). Various sequences of fabric layers were tested for composite air permeability following ASTM D737. Since no minimum air permeability threshold has been established for cloth face coverings for children, sequences with the highest composite air permeability were narrowed down, and polyester interlock knit (TestFabrics, Inc) reinforced with Pellon EK130 Easy-Knit™ (JoAnn Stores, Inc.) and OlyFun polypropylene spunbond nonwoven (JoAnn Stores, Inc.) fabrics were selected to compare against the Mayo Clinic recommended three layers of tightly woven cotton (TestFabrics, Inc). All fabrics were used as received. A single-use procedure mask recommended for ages 4–12 (O&M Halyard, Inc.) was included as a positive control. Two face mask designs were included: (1) “Lobster Tail” prototype developed in the previous study (Du Puis et al., 2022) and (2) Mask F, which was determined as providing the best fit among the commercial face masks tested in Design Exploration (for images see Appendix). The combinations of material and design for the constructed masks and the material specifications for the chosen fabrics are listed in Tables 2 and 3**,** respectively.

**Table 2:** Combinations of material and design for the constructed experimental face masks

Mask ID	Mask name	Material	Design
1	Surgical mask	PP2	Pleated
2	Cotton shaped	C/C/C	Mask F
3	Mixed shaped	PET/PP1/PET	Mask F
4	Mixed prototype	PET/PP1/PET	Lobster Tail

PP2 is multiple layers of polypropylene nonwoven, PP1 is single layer of polypropylene nonwoven, C is cotton plain weave, and PET is polyester interlock

**Table 3:** Material specifications of experimental face masks, Mask F and Lobster Tail

Material	Fiber content	Fabric structure	Mean fabric weight (gsm)	Mean fabric thickness (mm)	Mean TPI (warp/wale, weft/courses)
C	Cotton	Plain weave	119	0.19	142, 70
PET	Polyester	Interlock knit	101	0.28	39, 36
PP1	Polypropylene	Nonwoven	65	0.38	N/A

*PP1* single layer of polypropylene nonwoven, *C* cotton plain weave, *PET* polyester interlock

### Exhaled aerosol capture via simulated breathing apparatus (SBA)

**Fig. 1:**
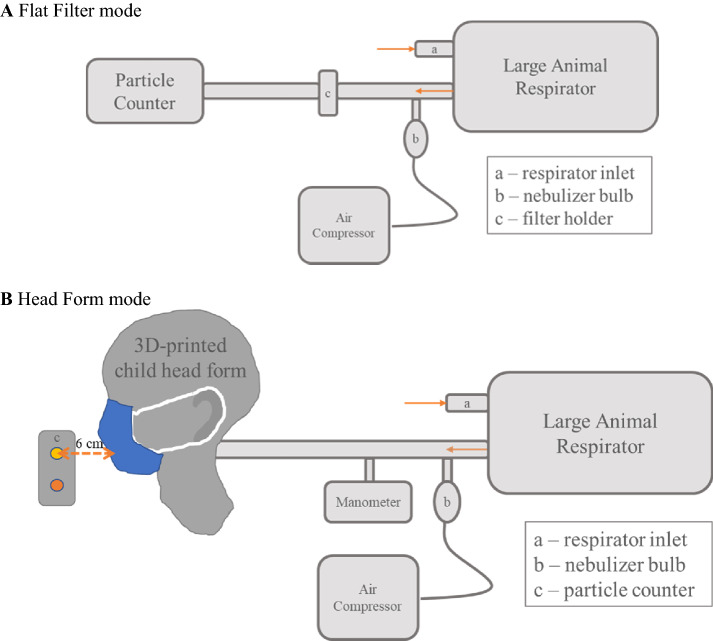
Simulated breathing apparatus schematic. **A** Flat Filter mode. **B** Head form mode

The experimental set up for evaluating particle capture in simulated breathing for is shown in Fig. 1A for flat textiles and Fig. 1B for masks on a head form. Commercial face masks were tested in Flat Filter (FF) Mode only. Experimental face masks were tested in both FF and Head Form (HF) Modes. All samples were measured in triplicate. Descriptive statistics were calculated as the mean and standard error throughout.

Briefly, a respirator pump (Harvard Apparatus, Model #B-55172) joined with a nebulizer bulb at a tee to the main hose line. The respirator pump was set to 200 ml tidal volume, 25 breaths/minute, and 50/50 inspiration/expiration ratio. The nebulizer bulb contained 1 M NaCl solution and was aerosolized by an air compressor (Medline Aeromist Plus Nebulizer Compressor, Model #MEDHCS60004). The salt aerosol was measured by a particle counter (MetOne Instruments, Aerocet 531S). High resolution channels on the particle counter were chosen as 0.3 μm, 0.5 μm, 1.0 μm, 5.0 μm, and 10.0 μm and scanned for 60-s increments with no hold. Polydisperse NaCl aerosol is a common challenge aerosol in filtration studies, and capture efficiencies of the 0.3 μm, 0.5 μm, and 1.0 μm particle sizes are within the Most Penetrating Particle Size (MPPS) size range (i.e., the intermediate particle size region with minimum filtration efficiency) (Konda et al., 2020; Lee & Liu, 1980; Rengasamy et al., 2010, 2017; Reyes & Frey, 2017; Zangmeister et al., 2020; Zhao et al., 2020).

The filtration efficiency for each size channel was calculated using Eq. 1:

1 $$Filtration \,efficiency, FE \left(\%\right)=\frac{Blank-Sample}{Blank}*100$$

where "Blank" is the average salt particle counts at the corresponding size channel without a sample in the filter holder (FF Mode) or mask on the head form (HF Mode), and "Sample" is the average salt particle counts at the corresponding size channel with a sample in the filter holder (FF Mode) or either the sealed or unsealed mask on the head form (HF Mode).

Commercial face mask samples were stored at 20 ± 2 °C and 25 ± 2% relative humidity. Experimental face mask samples were stored at 20 ± 2 °C and 50 ± 1% relative humidity. Within the range of relative humidity recorded, no effects of relative humidity were identified. Samples were weighed before testing (*Wix*), directly after testing (*Wwx*), and after 60 min in 60 °C oven (*Wdx*), where x denotes the individual layers for FF Mode and sealed or unsealed mask for HF Mode. Moisture content and salt content were calculated using Eqs. 2 and 3, respectively.

2 $$Moisture\, content \left(\%\right)=\frac{{W}_{wx}-{W}_{dx}}{{W}_{ix}}*100$$

3 $$Salt \,content \left(\%\right)= \frac{{W}_{dx}-{W}_{ix}}{{W}_{ix}}*100$$

Moisture capture efficiency and salt capture efficiency were calculated using Eqs. 4 and 5, respectively.

4 $$Moisture \,capture \,efficiency \left(\%\right)=\frac{{W}_{wx}-{W}_{dx}}{\rho Ft}*100$$

5 $$Salt \,capture\, efficiency \left(\%\right)= \frac{{W}_{dx}-{W}_{ix}}{CFt}*100$$

where ρ is the density of water, F is the averaged flow rate of aerosol, C is the concentration of salt in the aerosol (w/v), and t is sampling time. Sampling time is 10 min and 30 min for FF Mode and HF Mode, respectively.

#### Flat Filter mode

FF samples were cut into 38 mm circles and mounted into a filter holder downstream from the nebulizer bulb. Commercial face mask samples were assembled in their original sequence of layers. Uncaptured salt aerosol traveled through an inline moisture filter (Whatman cellulose filter paper type 4) before reaching the particle counter. Each sample was run with and without a sample (denoted with sample I.D. and blank, respectively) in the filter holder for 10 min per cycle.

#### Head Form mode

A representative child’s head scan was chosen from the Size North America body scan data of the 44 children who were 6 years old. The digital file was selected based on how well the ears were captured during data collection so that the experimental masks would stay firmly on the head form. The object (.obj) file was processed in Geomagic and MeshLab to create a 12.7 mm thick shell for 3D printing. Additionally, holes were cut in the shell to accommodate for nostril and mouth openings, along with a circle cut into the back of the head to allow for hose insertion for the apparatus. The dimensions were 5 mm diameter for the nostrils, 5.6 mm by 35 mm for the mouth, and 50.8 mm at the back of the head. The scan was divided into two parts vertically for printing and fastened with Velcro to allow for the insertion of the breathing simulator tubing. The head form was printed on a Stratasys Fortus mc400 3D printer with ABS m30.

The main hose line connected to a child’s mouthpiece (Voldyne Volumetric Exerciser kit, Teleflex Medical, Amazon.com) that was inserted through the back of the head, met flush with the mouth opening and sealed with silicone sealant. Pressure was monitored by a manometer (8205 Handheld 5 psi Digital Differential Pressure). Masks were mounted onto the child’s head form and sealed around their perimeter with two layers of Pro Gaff® gaffers tape, a heavy-duty cloth-based adhesive tape. Each cycle was run with no mask, unsealed mask, and sealed mask (denoted as such) for 30 min per mask sample. The particle counter was positioned at the level of the head form’s mouth for the first 15 min to quantify the through-mask particle penetration and at the level of the head form’s eyes for the last 15 min to quantify the around-mask leakage per mask sample. Particles were allowed to settle or disperse between samples to ensure the ambient particle count baseline was re-established before the next sample.

## Results

For all masks evaluated, filtration efficiency was consistently reported as 100% for the larger particles (5.0 μm and 10.0 μm), and therefore was not included in the analysis.

### Commercial face masks

#### Filtration efficiency and air permeability

**Fig. 2:**
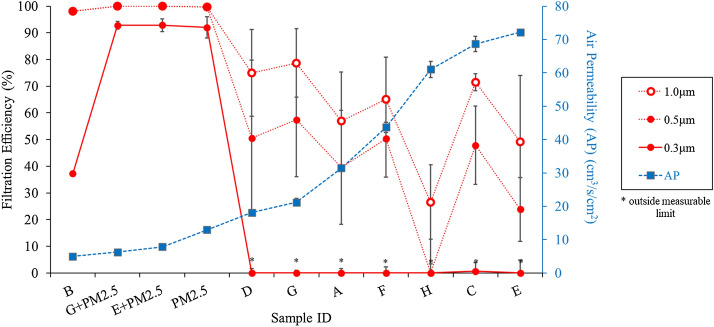
Commercial face masks evaluated via Flat Filter mode: filtration efficiency for 0.3 µm, 0.5 µm, and 1.0 µm particles and air permeability of combined mask layers

Filtration Efficiency and Air Permeability are ordered and plotted based on increasing composite air permeability in Fig. 2.

As expected, air permeability and filtration efficiency were inversely related and depended on various material factors such as fiber content, fabric structure, fabric weight, thickness, and thread count. Only masks containing PM 2.5-micron filters effectively captured 0.3 µm particles. These samples were also > 90% effective at all particle sizes; however, they had low air permeability. Adding the PM2.5 filters to Masks E and G increased the capture of all particle sizes but decreased the air permeability by 89% and 71%, respectively. Sample B was the only other face mask that captured any 0.3 µm particles. This mask with three layers of cotton poplin was also very effective at capturing larger particles but had the lowest breathability overall and much lower 0.3 µm capture efficiency compared to the PM2.5 filter samples.

For all other samples, regardless of specific fabric structure, negligible amounts of 0.3 µm particles were captured. Capture of 0.5 µm and 1.0 µm particles by non-PM2.5 masks had a weak correlation with breathability. Decreasing from 99% filtration efficiency of 1.0 μm particles in sample B to < 30% in sample H, the lower the airflow resistance, the smaller number of particles that were captured. While the recommended minimum air permeability was set for adult masks as 19.0 cm^3^/s/cm^2^ and a minimum has not yet been set for children, the child’s minimum can be expected to be higher relative to the adult’s minimum (American Association of Textile Chemists and Colorists, 2020). Masks A, C, E (without PM2.5 filter), F, and H surpassed this air permeability minimum but compromised their filtration efficiency.

#### Moisture content and capture efficiency

**Fig. 3:**
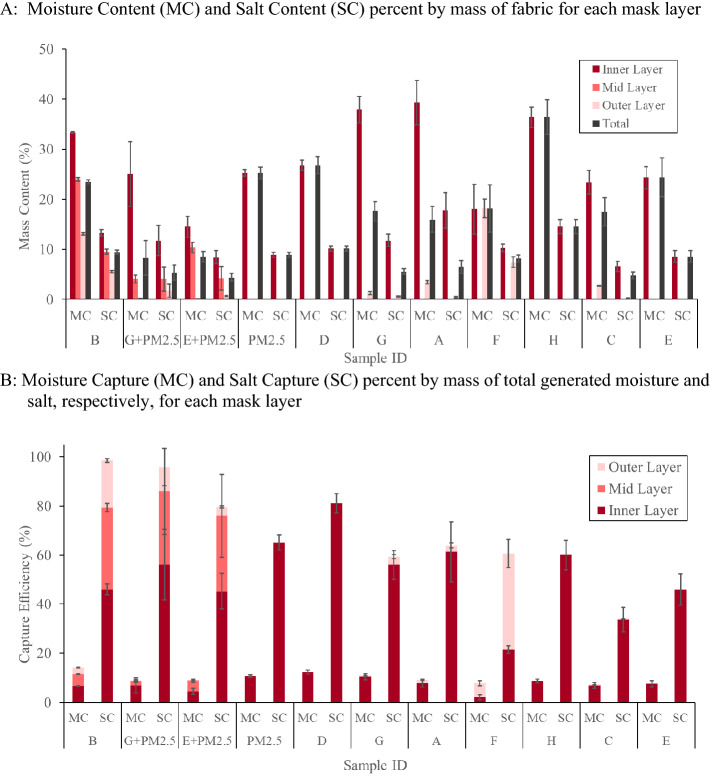
Commercial face masks evaluated via Flat Filter mode. **A** Moisture content (MC) and salt content (SC) percent by mass of fabric for each mask layer. **B** Moisture capture (MC) and salt capture (SC) percent by mass of total generated moisture and salt, respectively, for each mask layer

In FF testing, all material stacks from commercial masks had low moisture content (Fig. 3A), which was dependent on the fiber content of the inner layer. Material stacks with hydrophilic cotton fibers in the inner layer (Mask B, C, G and H) had the highest moisture content except for Mask C which had similar moisture content to stacks with hydrophobic fiber contents. Additionally, Mask A did not follow this trend as it had a hydrophobic inner layer but had similar moisture content to the masks with hydrophilic inner layer.

Moisture capture efficiency (Fig. 3B) was more dependent on the measured air permeability of the fabric structure and thread count of the fabric layers than on fiber content of the fabric layers. For example, Mask B had the highest moisture capture efficiency for total assembly, as expected, due to the combination of low air permeability and hydrophilicity of its three layers of tightly woven cotton. However, Mask D also had high moisture capture efficiency based on low air permeability despite its hydrophobic fiber content.

#### Salt content and capture efficiency

The salt capture efficiency (Fig. 3B) was found to be weakly related to the air permeability of the masks. Lower air permeability tended to translate to higher salt capture; Mask B had the lowest air permeability of the sample set and exhibited 98.5 ± 4.5% salt capture efficiency. The tighter structure of the low breathability fabrics allows for more contact area to trap and retain particles. Highly breathable fabrics, such as Mask E, on the other hand have larger inter-yarn pores that allow particles in the air stream to flow around the yarns and pass through the fabric. Mask C is an example of the value of coupling the particle count filtration efficiency and gravimetric salt capture efficiency. Mask C follows the CDC recommended number and composition of layers: three layers with the middle layer being nonwoven polypropylene and the remaining layers being woven cotton or polyester (Centers for Disease Control and Prevention, n.d.). Although Mask C exhibited similar filtration efficiency to the other non-PM2.5 samples, the salt capture efficiency was the lowest of the sample set at 34.0 ± 4.8%. Thus, Mask C was seemingly not retaining all of the particles it initially captured. Similar to the moisture capture results, Mask F also trapped more salt in the outer layer than inner layer. Since the commercial masks do not allow for well controlled variables, the results of this section are limited to the specific combinations’ success or failure. Material effects are explored further in a more controlled manner in the experimental face mask section.

### Experimental face masks

#### Flat Filter mode

To compare the experimental face mask samples to the commercial face mask samples, the fabric combinations in the experimental face masks were tested in the FF mode and for Air Permeability in Table 4.

**Table 4:** Material layering combinations evaluated via SBA Flat Filter Mode

Material	Air Permeability (cm^3^/s/cm^2^)	Filtration efficiency (%)			Moisture content (%)	Moisture capture efficiency (%)	Salt content (%)	Salt capture efficiency (%)
		0.3 µm	0.5 µm	1.0 µm				
Cotton (C/C/C)	2.61 (0.08)	15.6 (8.7)	80.6 (4.1)	90.1 (1.9)	56.4 (3.0)	1.1 (0.1)	13.2 (1.8)	4.4 (0.3)
Mixed (PET/PP1/PET)	45.14 (1.87)	0*	66.9 (3.8)	84.3 (2.1)	153.7 (12.7)	31.2 (2.9)	26.6 (3.5)	92.2 (7.7)
Surgical Mask (PP2)	47.08 (1.11)	60.8 (8.7)	98.3 (0.2)	99.6 (0.1)	93.2 (54.9)	3.5 (1.9)	22.2 (4.3)	12.8 (1.4)

Standard errors are in parentheses

*PP1* single layer of polypropylene nonwoven, *PP2* multiple layers of polypropylene nonwoven, *C* cotton plain weave, *PET* polyester interlock

*Outside measurable limit

The procedure mask samples had the highest air permeability and filtration efficiency for all particle sizes compared to the cotton and mixed samples. Cotton had higher filtration efficiency but critically lower air permeability than the mixed samples. The mixed samples had the highest mass content and capture efficiencies with procedure masks next and cotton lowest. The cotton combination had similar results to Mask B (also three layers of tightly woven cotton) except that the experimental cotton had low salt capture efficiency and Mask B approached 100% salt capture efficiency, which could possibly be attributed to the differences in fabric finishes if any. The mixed combination with a permanent nonwoven as its mid layer achieved a balance between air permeability and filtration efficiency by emulating Mask F with its lightweight, hydrophobic layers and Mask G with the multilayer, removable PM2.5 filter as its mid layer. Due to its low fabric weight, the mixed combination had higher salt and moisture mass content than any of the commercial masks but had similar capture efficiencies as Mask G with its PM2.5 filter.

#### Head Form mode

**Fig. 4:**
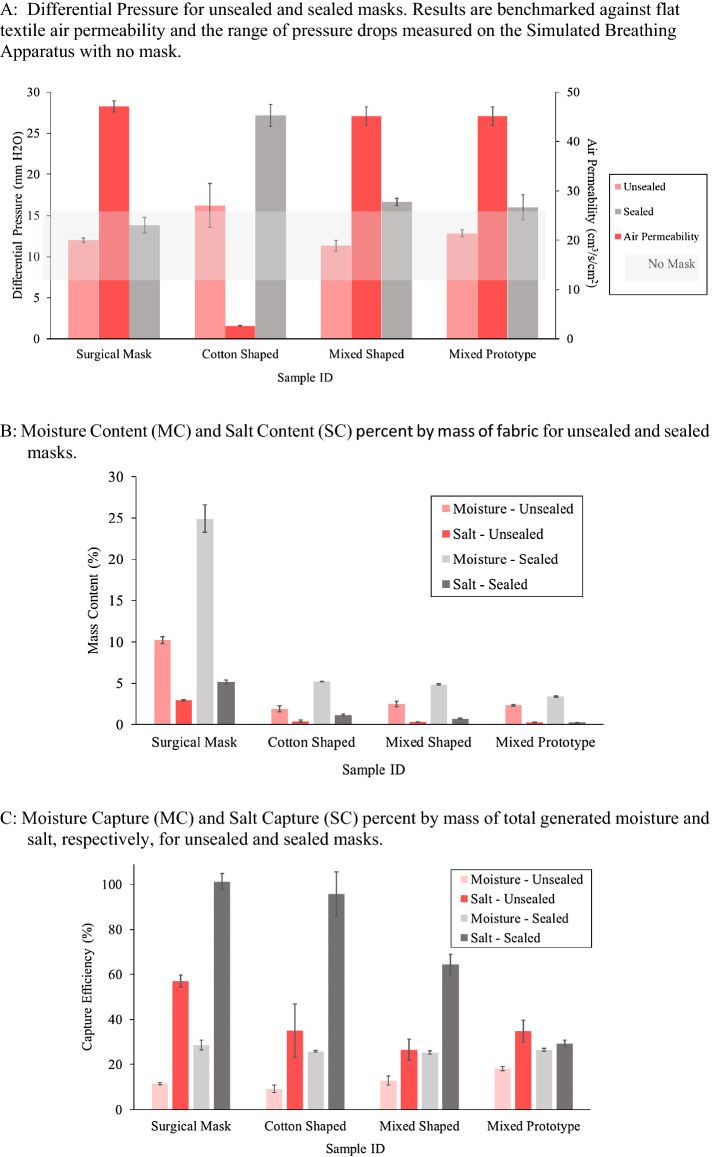
Experimental face masks evaluated via Head Form mode. **A** Differential pressure for unsealed and sealed masks. Results are benchmarked against flat textile air permeability and the range of pressure drops measured on the simulated breathing apparatus with no mask. **B** Moisture content (MC) and salt content (SC) percent by mass of fabric for unsealed and sealed masks. **C** Moisture capture (MC) and salt capture (SC) percent by mass of total generated moisture and salt, respectively, for unsealed and sealed masks

Over the test duration, no increase in pressure was observed for the unsealed trials, shown in Fig. 4A. Since differential pressure is a function of air flow rate and resistance to flow of the filter media, the invariant differential pressure means that the resistance to flow through the mask was either, (1) unaffected and the salt accumulation was not sufficient to block air flow or (2) affected and to maintain constant pressure, either the flow rate decreased over the sampling time or the aerosol must be forced around the mask instead of through the fabric layers. Sealing the masks forced the aerosol to only penetrate the mask and prevented leakage around the sides of the mask. In the sealed mask trials, the differential pressure still did not increase over the sampling time. Therefore, the accumulation of salt particles and moisture (Fig. 4B and C) was not sufficient to impede airflow, and the differential pressure was comparable to the initial mask air permeability.

##### Mouth Level FE

Masks with their corresponding filtration efficiencies are listed in Table 5. All mask types had greater than 70% FE at 0.5 and 1.0 μm particles for both sealed and unsealed trials.

**Table 5:** Experimental face masks evaluated via Head Form Mode. Standard errors are in parentheses

Sample ID		Mouth level filtration efficiency (%)			Eye level filtration efficiency (%)		
		0.3 µm	0.5 µm	1.0 µm	0.3 µm	0.5 µm	1.0 µm
Surgical mask	Unsealed	84.8 (4.3)	96.0 (2.1)	96.2 (2.1)	1.2 (3.1)	0.90 (35.7)	− 3.9 (52.1)
	Sealed	71.7 (15.3)	98.8 (0.7)	99.5 (0.3)	21.5 (2.3)	89.1 (2.4)	95.1 (1.1)
Cotton Shaped	Unsealed	84.2 (2.7)	97.2 (0.9)	98.2 (0.8)	− 1.1 (2.6)	36.3 (21.5)	54.5 (22.9)
	Sealed	57.6 (6.6)	95.8 (1.0)	97.2 (0.9)	− 5.1 (3.4)	24.9 (17.0)	35.2 (21.0)
Mixed Shaped	Unsealed	75.7 (5.2)	98.0 (1.0)	98.5 (1.1)	6.6 (6.1)	20.8 (32.7)	23.2 (51.7)
	Sealed	35.6 (2.8)	93.1 (1.1)	96.7 (0.5)	13.6 (7.2)	80.7 (8.6)	91.1 (3.6)
Mixed Prototype	Unsealed	36.8 (12.0)	87.2 (2.7)	87.0 (2.0)	− 5.3 (4.8)	− 96.1 (72.2)	− 206.5 (145.7)
	Sealed	13.4 (11.6)	71.6 (20.2)	72.3 (23.5)	20.8 (1.4)	88.7 (3.1)	90.3 (5.4)

Originally, we tested the hypothesis that unsealed masks would result in lower FE than the FF mode and that sealing the masks should result in FE approaching the FF mode values. The unsealed part of this hypothesis was proven false as the FE was actually higher than the FF mode due to design effects such as deadspace allowing for the velocity to decay before impacting the mask (Tcharkhtchi et al., 2021). The second part of the hypothesis was proven to be true. Sealing the masks did cause them to approach their FF mode values. However, instead of resulting in increased FE, sealing the masks reduced the FE as compared to the unsealed masks. As expected, sealing the masks increased the differential pressure for all mask types. The cotton mask exhibited the largest increase, as expected as it had the lowest air permeability. An alternate hypothesis is that the increase in pressure of sealing the mask translates to higher face velocity at the mask surface which has previously been shown to correlate to lower FE (Tcharkhtchi et al., 2021).

The cotton shaped mask exhibited similar FE at all particle sizes for unsealed trials as the procedure mask. However, the low breathability of the cotton shaped mask will make it uncomfortable to wear and encourages the exhaled aerosol to flow around the mask instead of through the mask. This leakage is reflected in the salt capture data. The mixed shaped mask had lower but still significant FE of 0.3 μm particles for unsealed trials compared to the cotton shaped and procedure masks. The mixed prototype mask had significantly lower FE at all particles sizes compared to the other mask types. While the mixed prototype mask had the lowest FE performance, it still had acceptable levels of FE for nonmedical mask use. The high breathability of the mixed shaped and prototype masks will make them more comfortable to wear and promotes the exhaled aerosol to flow through the mask rather than around the mask. While the salt capture efficiency of the mixed prototype is lower than the other masks, it does not change when the mask is sealed. This invariance to sealing corresponds to the mixed prototype’s performance being optimized to the material’s maximum performance. For the other masks, sealed versus unsealed performance leaves an opportunity gap for their material or design *choice* to be further optimized.

##### Eye Level FE

The particle counts collected at eye level are a combination of aerosol leaking out the top of the mask as well as particles penetrating the mask and diffusing as a plume. Therefore, FE less than the corresponding mouth level or negative FE correlate to leakage out the top of the mask. Unlike the mouth level FE, only the sealed procedure mask, mixed shaped, and mixed prototype samples had greater than 70% FE at 0.5 and 1.0 μm particles. Since the eye level particle counts are not collected in plane with the aerosol source as the blank and mouth level particle counts are, the sealed masks at eye level did not match the unsealed or sealed masks at mouth level. All unsealed masks had poor FE and high standard errors, correlating to high amounts of leakage out the top of the mask and large variations in mask performance even with proper and consistent donning. The mixed prototype had the highest amount of leakage, attributed to the bulkiness of the fabric, seams, and silicone elastic at the bridge of the nose. The nose wire is relatively weak, so any bulkiness inhibited the seal at the top of the mask. Thus, the discrepancy in mouth and eye level FE results informed the design features’ effectiveness and can be used to improve the next iteration of the mask design.

## Discussion

Assessing the effectiveness of a mask’s design and material choice requires multiple performance metrics that consider the breathability, aerosol capture, and comfort of the mask. Air permeability is a useful static measurement that correlates well to sealed masks. In unsealed masks, aerosol follows the path of least resistance, which in low air permeability masks will be through the gaps at the mask edges rather than through the mask layers. Higher air permeability minimizes leakage while conceding only marginal particle penetration in multilayer mask configurations.

The combination of moisture and salt capture performance can provide a preliminary prediction of the effectiveness of the stacked fabric layers used in face masks. Masks need to be selectively permeable to moisture but impermeable to aerosolized particles to maximize the filtration effectiveness without impeding the wearer’s comfort. The moisture content of the inner layer is pertinent as it resides next to the face, which has the largest effect on the perception of dryness and therefore, has the largest effect on the wearer’s comfort. With functional apparel, the goal is commonly to maintain a dry environment in the microclimate between fabric layer and skin (Watkins & Dunne, 2015). That is, the more moisture that is retained, the heavier the fabric is and the higher the wearer discomfort. Moreover, too high humidity in the deadspace and warm moisture collected in the mask allow for microorganisms to stay alive and spread to other parts of the mask (Tcharkhtchi et al., 2021). In conflict with this goal, moderate humidity within the mask is beneficial to the protective function of the mask to hydrate inspired dry air (Courtney & Bax, 2021). To optimize humidity within the mask while maintaining dryer fabric next to the wearer’s face, the mask assembly should be composed of a hydrophobic inner layer with low moisture content and a hydrophilic mid layer with high moisture absorption. Mask F demonstrates this layering and low overall moisture capture efficiency. The low thread count of the highly breathable, hydrophobic inner layer allows for higher transmission of moisture through the inner layer and capture by the hydrophobic outer layer with much lower air permeability. Separation of a single layer mask into multiple layers decreases the moisture retention in the inner layer against the wearer’s face and increases the overall comfort of the wearer. Another solution is to introduce a PM2.5 filter to the mask assembly. Including the PM2.5 filter in the Mask E and G assemblies decreased their inner layer and total moisture contents without critically affecting their total moisture capture efficiencies. The PM2.5 also appeared to wick some moisture away from the inner layer, potentially improving the comfort of the overall mask.

The effectiveness of the fabric layers as filters for aerosolized particles is determined by counting the number and size of particles that penetrate the fabric, quantified as the filtration efficiency, and the total mass of salt captured during the 10-min testing time. Particles larger than 1.0 μm are captured by all fabrics with 100% filtration efficiency; however, the total mass of salt captured does not discriminate the particle sizes captured. Therefore, high filtration efficiencies for particles less than or equal to 1.0 μm do not necessarily correlate to higher salt mass capture. Further, capture of MPPS particles (0.3 μm, 0.5 μm, and 1.0 μm) is critical for source control of particles that could contain and transmit pathogens. These particles should be trapped by a mid layer. Particles captured by the outer layer have been shown to have the potential for contaminating surfaces that the mask may come in contact with and transfer to, or leaking particles dislodged by the breathing force to the environment (Tcharkhtchi et al., 2021). Therefore, adding a middle layer, whether permanent or temporary (i.e., PM2.5 filter), can minimize the particles reaching the outer layer. Both Mask E and G with the PM2.5 filter inserted and Mask B with a permanent middle layer exhibit high salt capture efficiency for the inner and mid layers with proportionally smaller salt capture efficiency of the outer layer. These masks had the best filtration performance overall with the only tradeoff being poor breathability. As suggested before, we propose that this tradeoff can be minimized by optimizing the fabric composition and structure parameters of the mask layers as a system.

Evaluating the prototype mask in its constructed configuration on the head form allows for simulated wear results that can be used in the iterative design process to improve the design considerations towards a comfortable and effective mask design. An example was the silicone elastic that was added around the perimeter of the pleated filtration panel in the prototype mask. The silicone elastic was chosen based on the designer’s functional apparel experience with garments that must not slip but also stretch with the wearer. Cloth face masks have poor sealing and require flexibility to accommodate jaw movement, so silicone elastic could lend itself well here. Any added benefit of the silicone was outweighed by the bulkiness of the silicone elastic on top of the fabric layers and seam choices. This bulkiness prevented the mask from conforming to the contours of the face, especially the nose bridge area. The poor fit led to considerable aerosol leakage, which is a safety issue as well as practical issue for users who wear glasses, as leakage out the top of the mask causes fogging (Du Puis et al., 2022). Because this element of the mask design was identified early in the design process, alternatives can be further explored in the next iterations.

This study has a few limitations. The commercial children’s face masks were only tested as flat textiles. Evaluating them as purchased in HF mode would give more information on their design effects as well as allow for comparison within the set and to the experimental face masks. We used a simulated breathing apparatus with a single rigid head form, which allows control over the experimental variables and returns objective results, but no perception threshold of comfort or subjective feedback is obtained. The single head shape and size is not representative of fit for other children’s head shapes and sizes, so the masks should be evaluated on additional child head forms. Further, the rigid plastic of the head form does not perfectly emulate the viscoelastic properties of facial skin, so the fit and seal of the face masks is different than with a more human-like silicone layer. The challenge aerosol was at room temperature which is lower than the temperature that exhaled breath would be exiting the mouth, so thermal comfort was not able to be evaluated. Thermodata sensors placed on the head form inside and outside the mask at specific sites can monitor moisture and temperature buildup during breathing as well as detect aerosol leakage (ie. eye, cheek, chin). Human subject testing with a PortaCount FE test and comfort testing could add more meaningful qualitative and quantitative feedback.

## Conclusions

Commercially available and experimental cloth face masks were studied for their material performance under conditions modeling breathing patterns of children 4 to 6 years old. Materials were evaluated with a simulated breathing apparatus using appropriate children’s breathing parameters as flat textiles and as constructed masks on a 3D printed child’s head form. The flat textiles followed the trend of lower breathability corresponding with higher filtration efficiency; however, only the single use procedure mask materials stack was found to have both high filtration and acceptable air permeability. Fabrics with hydrophilic fiber content and low air permeability captured the most moisture, which could become uncomfortable especially in the layer next to the face. Lower air permeability fabrics also tended to correlate to higher salt capture. Material stacks with one or two layers were insufficient as they held the captured aerosol next to the face while also contaminating the outside of the mask. Therefore, the recommended fabric layering system is heuristically defined as a hydrophobic, higher air permeable inner layer with low moisture content, an absorbent mid layer with moderate air permeability, and low air permeable outer layer.

The head form tests revealed that a higher air permeability of the multiple layers of synthetic knit and nonwoven fabric with a proper fitting mask is more effective overall than a tightly structured, poorly air permeable layering of cotton woven fabric. All experimental masks successfully captured > 70% of 0.5 µm and 1.0 µm particles at the level of the head form’s mouth. Leakage out the top of the mask was identified in all masks as evidenced by the filtration efficiency at eye level being significantly lower than at mouth level. The leakage can be reduced by proper seal of the mask by either reducing the bulk at the nose bridge or substituting the aluminum nose wire with a stronger material such as steel. Thus, face masks designed for children can be made from various combinations of materials depending on the mask pattern and desired performance metrics. This study underscores the importance of the material testing as a step in the design iteration process and shows how the materials testing results can inform areas of improvement in the next iteration of design. Findings and filtration test method developed from this study can also be applied to general face mask material selection and evaluation.

## Figures and Tables

**Fig. 5:**
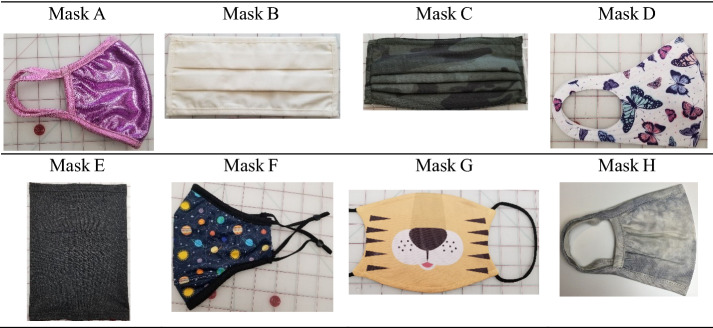
Commercial masks purchased for pilot wear trials

**Fig. 6:**
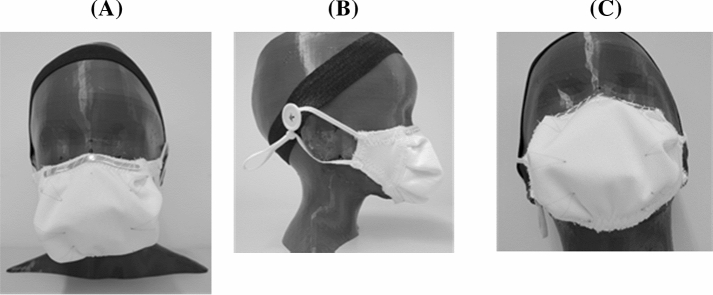
Lobster mask fit Front (**A**), side (**B**), detail on 3D printed child head form (**C**)

## Data Availability

all data is available via the Frey research lab and archived electronically on Cornell Box.

## Appendix

See Figs. 5 and 6.
